# Three Years of High Time-resolution Air Pollution Monitoring in the Complex Multi-source Harbor of New York and New Jersey

**DOI:** 10.4209/aaqr.2020.02.0069

**Published:** 2020

**Authors:** Gayle Hagler, Dan Birkett, Ronald C. Henry, Richard E. Peltier

**Affiliations:** 1 United States Environmental Protection Agency, Office of Research and Development, Research Triangle Park, NC 27711, USA; 2 United States Environmental Protection Agency, Region 2, New York City, NY 10007, USA; 3 Department of Civil and Environmental Engineering, University of Southern California, University Park, Los Angeles, CA 90007, USA; 4 Department of Environmental Health Sciences, University of Massachusetts, Amherst, MA 01003, USA

**Keywords:** Sulfur dioxide, Nitrogen oxides, Black carbon, Port, Shipping

## Abstract

In densely developed port areas with numerous emissions sources, relating measured air quality changes to emissions is challenging given the geographic density of sources without unique pollutant composition signatures. To better understand air quality during increasing emission controls at the Port of New York and New Jersey (“Port”), an air monitoring station was sited to minimize collinearity of sources along ordinal directions. The study area includes an international airport, interstate highway, port terminals and shipping lanes, and industrial sources, as well as typical urban emissions of a megacity. Because air flow travel time from sources to the monitor were usually much less than one hour, minute-by-minute, high-precision data were collected for three years (2013–2015) for sulfur dioxide (SO_2_), carbon monoxide (CO), oxides of nitrogen (NO, NO_2_), black carbon (BC), fine particulate matter (PM_2.5_), and meteorology (wind speed, wind direction, temperature, humidity). From summer 2014 to spring 2015, hourly metals data were also collected. A high degree of temporal variability was observed for pollutants associated with direct emissions, with highest hourly average coefficient of variation observed for NO (2.65), SO_2_ (1.45) and BC (1.21). Nonparametric trajectory analysis (NTA) was utilized to separate the source areas influencing the monitoring data and observe how they changed over time, with over 1.6 million trajectories computed in total. Comparing the last 5 quarters of the study to the first 5 quarters, concentrations at the monitoring site associated with three port-related geographic areas decreased by 34–41%, 11–17%, and 28–41% for SO_2_, NO_x_, and BC, respectively. Over the same period, indicators of shipping and cargo activity at the port remained relatively constant; therefore, a shift in emission factors is likely the cause of the change. This study demonstrates the value of high-time resolution, accurate monitoring data along with careful siting to understand source area influences.

## 1 INTRODUCTION

Exposure to air pollution is an ongoing source of public health concern. In areas nearby emissions associated with transportation—such as highways, airports, ports, and railyards—local elevation in pollutants associated with direct emissions has been detected in downwind areas (Karner *et al.*, 2010; Zhu *et al.*, 2011; Steffens *et al.*, 2017; Brantley *et al.*, 2019). The geographic scale of impact has been shown to vary depending on local topography and meteorology, but the greatest impact is generally within 10 km. A growing variety of methods are being developed and implemented to gain further insights on dynamic trends associated with source emissions and local-scale meteorology, including low cost air sensors (e.g., Snyder *et al.*, 2013; Heimann *et al.*, 2015) and mobile monitoring (e.g., Apte *et al.*, 2017).

Changes in transportation-related air pollutant emissions affect both local-scale and regional-scale air pollution levels. Natural experiments—known events involving a shift in emissions—are opportunities to connect a change in emissions to the resulting shift in air pollution levels. The connection between a known emissions perturbation and measured air pollution concentrations is not always clear cut, as near-source air pollution concentrations depend upon the reactivity and lifetime of the particular pollutant of interest, the mixture of other pollutants in the atmosphere, meteorological conditions, and the degree to which other sources of emissions for the pollutant may have varied during the same time period. On a larger geographic scale, particulate matter (PM) composition along with Positive Matrix Factorization has been utilized to estimate PM reductions associated with emissions changes due to new regulations in the United States on marine vessel fuel sulfur content (Kotchenruther, 2015). At a near-source scale, typically under several km in distance from sources of interest, field study design often isolates a specific source through finding study locations that have minimal confounding sources in close proximity (e.g., Rizzo *et al.*, 2014). However, in locations such as a major port area, other transportation sources are often proximate to efficiently facilitate goods movement, complicating study design. When air monitoring and meteorological data are collected at a high time-resolution—on the order of one to ten minutes in duration—a number of analytical strategies can be employed to isolate how nearby sources may affect the monitoring data, particularly for pollutants produced by direct emissions. Strategies used in past studies include a simple assessment of concentrations binned by wind direction and speed, conditional probability functions relating wind direction and speed to concentration thresholds of interest (Ashbaugh *et al.*, 1985, Uria-Tellaetxe and Carslaw, 2014) and Nonparametric Trajectory Analysis (NTA) which extends beyond wind-directional analyses to reveal how surrounding geographic areas contribute to measured concentrations (Henry, 2008; Henry *et al.*, 2009; Henry et al, 2011).

**Fig. 1.**
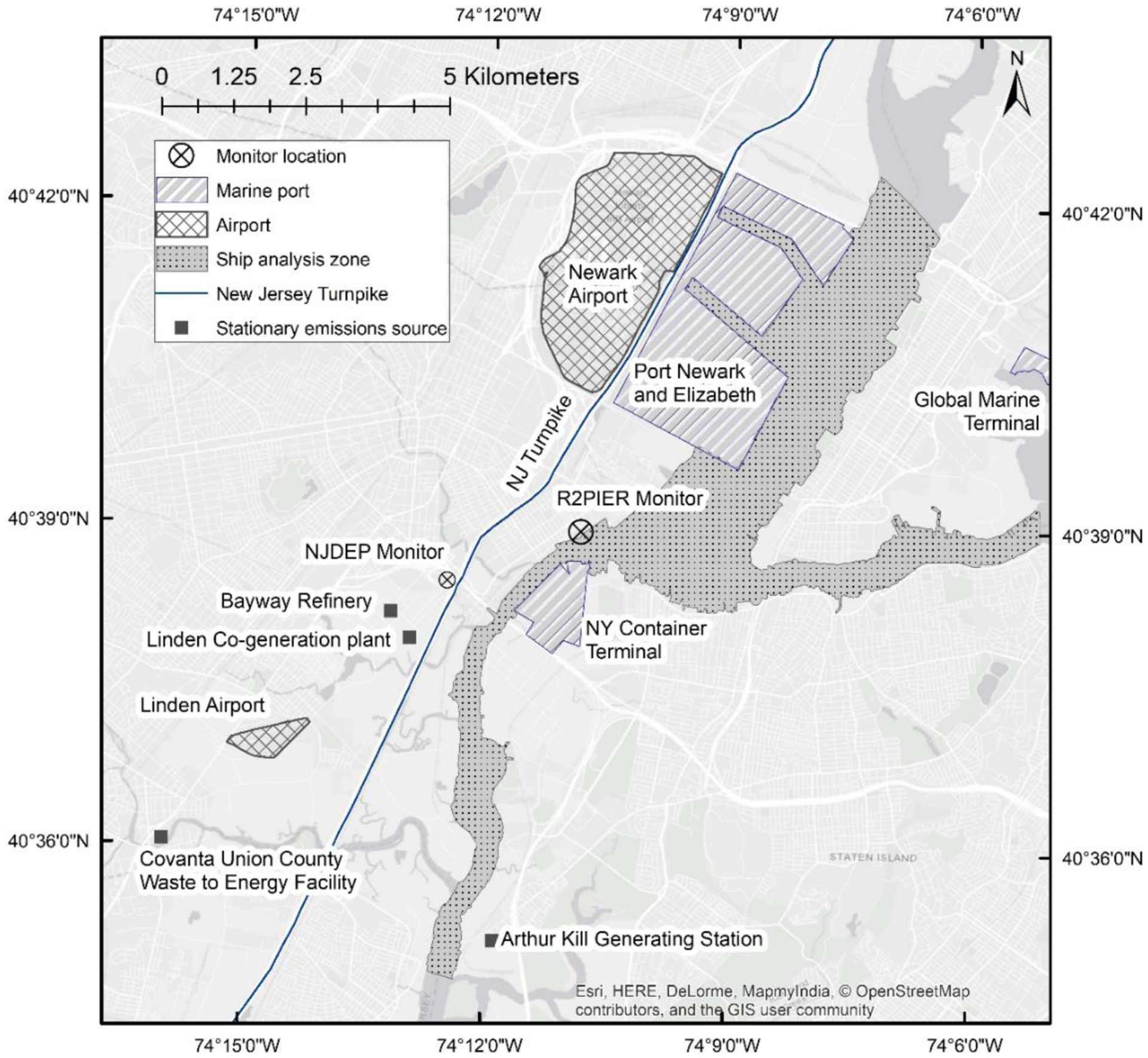
Field site location.

The study site of focus in this paper has, within a 10 km radius, a major port with multiple terminals, rail yards, refineries, a heavily used interstate highway, a busy international airport, shipping lanes in active use, as well as a power plant, see Fig. 1. In such an environment, connecting real-world measurement data to specific source areas, and known emissions perturbations, presents a significant challenge because of directional collinearity of sources and air flow travel times to the site that are usually much less than 1 hour.

The Port of New York and New Jersey, referred to hereafter as the Port, is one of the busiest ports in the United States. Growth in container traffic, measured by twenty-foot equivalent units (TEUs), grew by 15% between 2012 and 2015 (NJDEP, 2011; PANYNJ, 2018). Previous modeling studies by the state indicated emissions associated with the Port were a cause for increased health risk (NJDEP, 2009). While the report recommended an air monitoring study be conducted, it noted the difficulty in connecting monitoring data with a particular source given the challenge of uniquely identifying diesel exhaust associated with the Port from confounding sources.

Although the volume of TEUs at the Port has increased sharply over time, a number of emission reduction activities have taken place. Between 2012 and 2015, the Port Authority of New York and New Jersey’s emissions inventories estimated reductions in fine particulate matter of 38% and reductions in sulfur dioxide of 95%. Emissions of nitrogen oxides were estimated to increase over the same period by 2.4%. Longer term trends showed substantial reduction in nitrogen oxides (PANYNJ, 2019b).

A notable cause of air emissions change was the designation of the North American Emissions Control Area, which became enforceable in August 2012 and led to the reduction of allowable sulfur content in fuel oils used by ocean-going vessels (OGVs) to 1%, followed by a decrease to 0.1% starting in January 2015. Under a Clean Air Strategy, the Port Authority of New York and New Jersey undertook additional actions to reduce its air pollutant emissions, including replacement of trucks, fleet modernization of cargo handling equipment, and locomotive engine replacements. Replacement of older trucks was also accelerated as a consequence of damage to the Port area from Hurricane Sandy during the fall of 2012.

This paper describes a multi-year air monitoring study to observe air quality near the Port during this period of emissions transition. High time-resolution multipollutant and meteorology measurements were conducted during the study timeframe, creating a very large data set that spans several years and supporting the implementation of strategies such as NTA to discern how source emission areas impacted nearby monitoring site concentrations over time.

## 2 METHODS

### 2.1 Site Description

An air monitoring station was located strategically to minimize collinear sources, or conversely speaking, to maximize the ability for wind-directional analyses to associate measurements with nearby unique source areas. As shown in Fig. 1, the monitoring station (Latitude: 40.649019; Longitude: −74.180835) had major sources located at different compass angles, including several terminals to the Northeast and South, the Newark airport to the North, a major highway running to the North and West, shipping channels to the Northeast, East, and Southeast, and other industrial sources to the Southwest. Air monitoring instruments were utilized that provided trace-level pollutant detection (Table 1) as well as the ability to resolve air pollution levels at a 1-minute time resolution. Measured parameters included fine particulate matter (PM_2.5_, or particles with aerodynamic diameter smaller than 2.5 micrometers), black carbon (BC), sulfur dioxide (SO_2_), carbon monoxide (CO), and oxides of nitrogen (NO, NO_2_, NO_x_), wind speed and direction, temperature (T) and relative humidity (RH). These high time-resolution measurements were initiated in June 2012 and sustained through September 2015. An auto-calibration system was utilized to conduct daily zero and span checks on all gas analyzers. Zero and flow checks were conducted on the particulate matter instruments upon installation and after each maintenance event (e.g., filter tape change).

The PM_2.5_ instrument was a unique design, coupling a beta-attenuation mass measurement with optical detection via nephelometry. This detection approach took advantage of the real-time detection capability of optical methods, while providing a true mass measurement to calibrate the real-time signal. The instrument produced three data outputs, therefore—the mass concentration from the beta-attenuation detector, the estimated mass concentration from the nephelometer, and the combined signal referred to as the “SHARP” output. The SHARP data output was utilized for all analyses presented. At the outset of the study, the nephelometer component of the instrument malfunctioned and required repair, which led to a later start time for the PM_2.5_ data (December 2012). Further, the monitoring station was entirely powered down as a preventative measure during Hurricane Sandy and was fortunate to have sustained minimal damage. This preventative shutdown and the aftermath of the storm interrupted data collection beginning October 28, 2012 with gas monitors and wind data back operational on November 6 and the black carbon monitor operational on November 9. A second cause of data disruption was due to a deviation in performance of the black carbon monitor mid-way through the project, where the instrument’s data shifted to have increased noise in its output. The monitor was temporarily replaced with an identical version, while the original was repaired and then reinstalled. Beyond these interruptions, other causes for data gaps were temporary in nature and due to periodic instrument maintenance or troubleshooting. Completeness for each measure, on a quarterly basis, is summarized in Table 2. Beyond the high-time resolution monitoring data sustained for three years, hourly particulate matter metals data were measured for approximately nine months, June 2014-March 2015 using an XAct 625 (Cooper Environmental Services). The metals data averages are provided in Supplemental Information (Table S1) and specific parameters compared with a nearby monitoring site (Table S2), but not discussed in detail, with the focus of this paper being the longer-term high time resolution data.

### 2.2 Shipping and Cargo Data

In this paper, the term ship refers to large ocean-going vessels. These ships have engines greater than 30 liters per cylinder and are classified by EPA as category 3 marine vessels. The term cargo ship refers to the Automatic Identification System (AIS) ‘Type of Ship’ classification with a two-digit numeric code in the 70s. All cargo ships operating in the study area are believed to be category 3 marine vessels. Smaller category 2 and category 1 marine vessels, like tugs and ferries, are covered under EPA’s 40 Part 80 Clean Air Act fuel ultra-low sulfur diesel 15 ppm fuel sulfur limit. The AIS data disseminated by the U.S. Coast Guard was utilized to estimate shipping activity near the Port during the study period. The AIS data include information on vessel location, size, type, speed, and heading. Data for the entire study period were obtained from the U.S. Coast Guard in a pre-processed and summarized format at a five-minute resolution. The data were further processed to remove errors such as duplicate entries and invalid vessel locations (e.g., on land), to remove incomplete entries, and exclude vessel locations beyond an 8 km radius, while including the main ship channel, the Kill Van Kull between New Jersey and Staten Island.

The AIS data were grouped according to vessel type and speed values reported in the AIS messages. Vessels with speeds below 0.2 knots were assumed to represent ships in hoteling mode at berth, in which emissions result from the use of auxiliary engines and boilers, but not main propulsion engines. For general tracking of activity, the metric evaluated was vessel “ping,” or record of activity, indicating the presence of a vessel in the Port area for each five-minute interval throughout the study period. Vessel modes were categorized based on speed as either stationary hoteling mode or in transit. The study focused on cargo ships, where allowable fuel sulfur levels dropped to 0.1 percent in January, 2015 from 1.0 percent prior to this time period. In addition, monthly cargo volumes, measured as throughput of twenty-foot equivalent units (TEUs), published by the Port Authority of New York and New Jersey (PANYNJ, 2019a) were used as a general indicator of port activity.

### 2.3 Data Analysis Approaches

Initial processing of the air monitoring data was conducted using MATLAB version 2014b, including concatenating monthly files and removing any data flagged as associated with calibration checks. In total, the volume of minute-by-minute data approximated 1.7 million observation times over the 3+ years. For the gas-phase instruments, a secondary check was conducted to flag any significant negative values (below −2 ppb). In total, this led to a removal of 0% of data points for SO_2_, 0.0006% for NO_x_, and 0.009% for CO. Further, the black carbon data was initially processed using a MATLAB version of the optimized noise-reduction averaging (ONA) method with a minimum attenuation (ATN) change threshold of 0.05 (Hagler *et al.*, 2011). The original data set had 7.4% values below zero, which were a function of the high time resolution sampling rate (1 minute) and periods of low concentrations measured. The ONA algorithm conducts adaptive averaging along the time series based upon the ATN signal reported by the instrument. The result of the processing was that the original 1 minute data were now smoothed, with the average window size of ~4.5 minutes, but the variable window means that ~27% of the data were retained at their original timebase and 60% of the data had an averaging time period under 3 minutes, whereas about 5% of the data had averaging period exceeding 14 minutes (Fig. S1). After the ONA algorithm was utilized, ~2.7% of the BC data values were below zero and withheld from the analyses. These remaining negatives mostly corresponded to the timeframe when the BC monitor began exhibiting increased noise and needed repair, or rarely when a filter change occurred before the ONA ATN threshold was reached. In addition, the PM_2.5_ SHARP data were inspected for periods when cold temperatures caused the internal tape to fail to rotate correctly, causing disruption in sampling. This event primarily took place during January 2014. After visual inspection, a key indicator was a very low negative value reported by beta-attenuation monitor (BAM) component of the instrument. For periods when the BAM reported a value below −70 μg m^−3^, two hours prior to this data point and 24 hours of data afterward were set to missing. As a result, a total of 1.9% of the data were flagged and removed from analysis. After this initial processing, data were imported and analyzed using R version 3.5.1, with packages including gdata, openair, data.table, and plyr.

NTA analysis was conducted via MATLAB, using the one-minute monitoring data at the R2PIER site and meteorological data at a Newark Airport (KEWR) as inputs. NTA calculated local back-trajectories per minute using inverse distance-squared weighting interpolation of the wind data at the R2PIER site and KEWR Automated Surface Observation System (ASOS) 1-minute wind data. The ASOS wind data are two-minute averages reported every minute from a cup anemometer while the R2PIER data were produced by an ultrasonic 3-D anemometer. The wind speed and direction at a point was determined as the inverse-distance-squared weighted average of the wind velocity components from these two sites. The equations for the trajectory and other NTA calculations are detailed in Henry (2008) and most recently in Henry *et al.* (2019).

## 3 RESULTS AND DISCUSSION

### 3.1 Study Sampling Conditions and Overall Averages

**Fig. 2.**
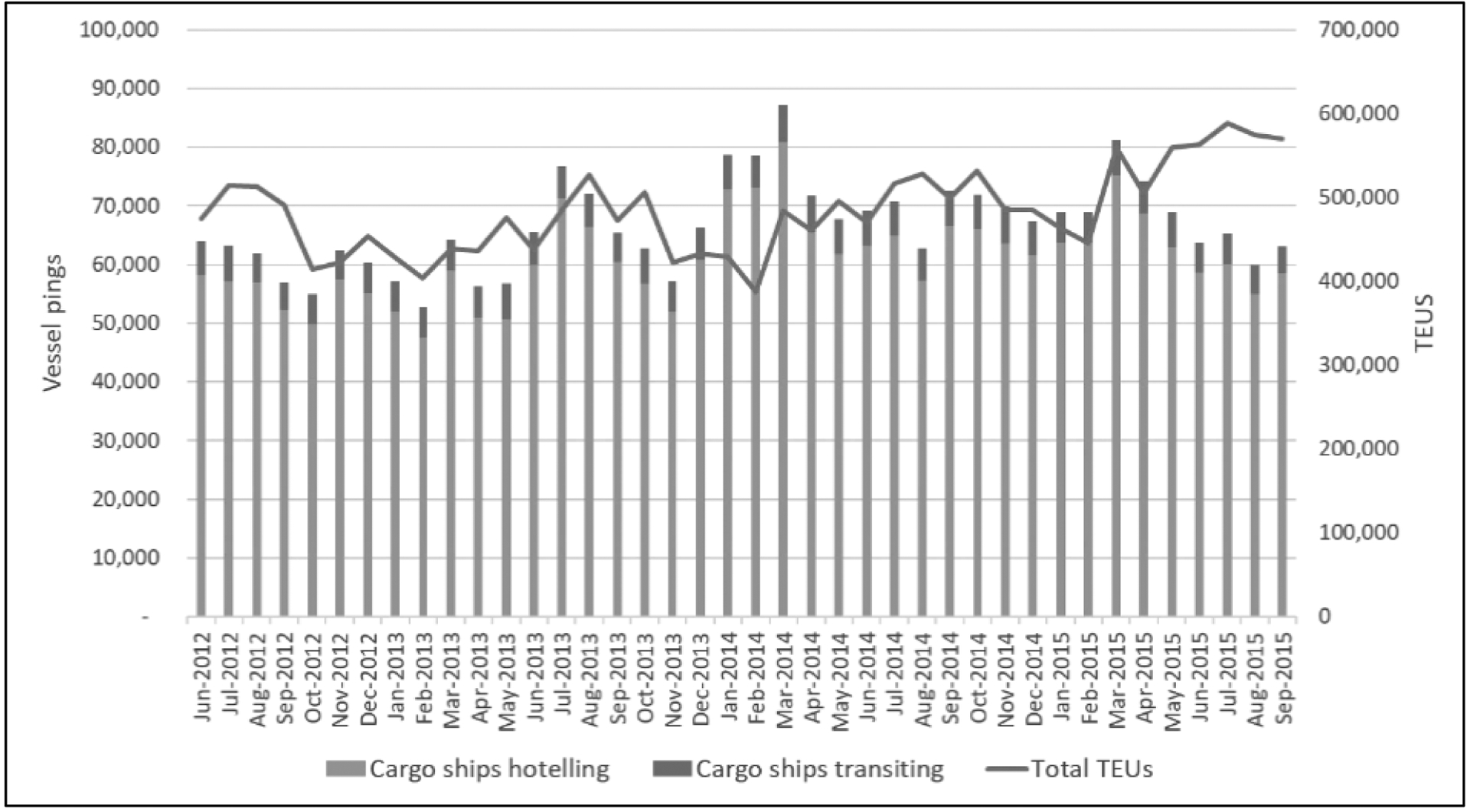
Shipping activity near the Port during the study period, where a “ping” indicates a recorded presence of a vessel and TEUs (twenty-foot equivalent units) indicates container throughput, a measure of port activity.

Shipping activity data fluctuated throughout the study, with TEUs and cargo ship number not consistently aligned (Fig. 2). The spring to summer of 2015 indicates an overall increase in TEUs relative to previous years, however the number of vessels did not increase in proportion to the TEUs. In general, vessel activity near the Port was dominated by hoteling operations, while in-transit operations comprised a small percentage of activity. The wind conditions throughout the sampling study are an important consideration to the connection of the high time resolution monitoring data to nearby source areas. Prevailing winds were typically from the west, ranging from the SW to NW, with wind speed between 2–4 m s^−1^. However, over each calendar year sampled, monitoring data were collected over all wind angles, with the high time resolution measurements equating to thousands of observations for any given 10-degree wind angle. An example showing data collection frequency by wind speed and direction for the four complete summer quarters is provided in Fig. S2. Summary data on pollutant concentrations and meteorology, as well as by season, are provided in Table 2. Seasonality is evident for all parameters measured, with maximum concentrations occurring during fall and winter months (October through March) and lower concentrations in the summer.

Comparing this location with other areas in the United States for the years 2012–2015, via the U.S. EPA’s trends reports (https://www.epa.gov/air-trends), the CO data measured in this study was below the 10^th^ percentile of 44 Trend Sites reported nationally, NO_2_ values were similar to the 10^th^ percentile value, SO_2_ values were well below the 10^th^ percentile, and PM_2.5_ levels were slightly below the 90^th^ percentile.

**Fig. 3.**
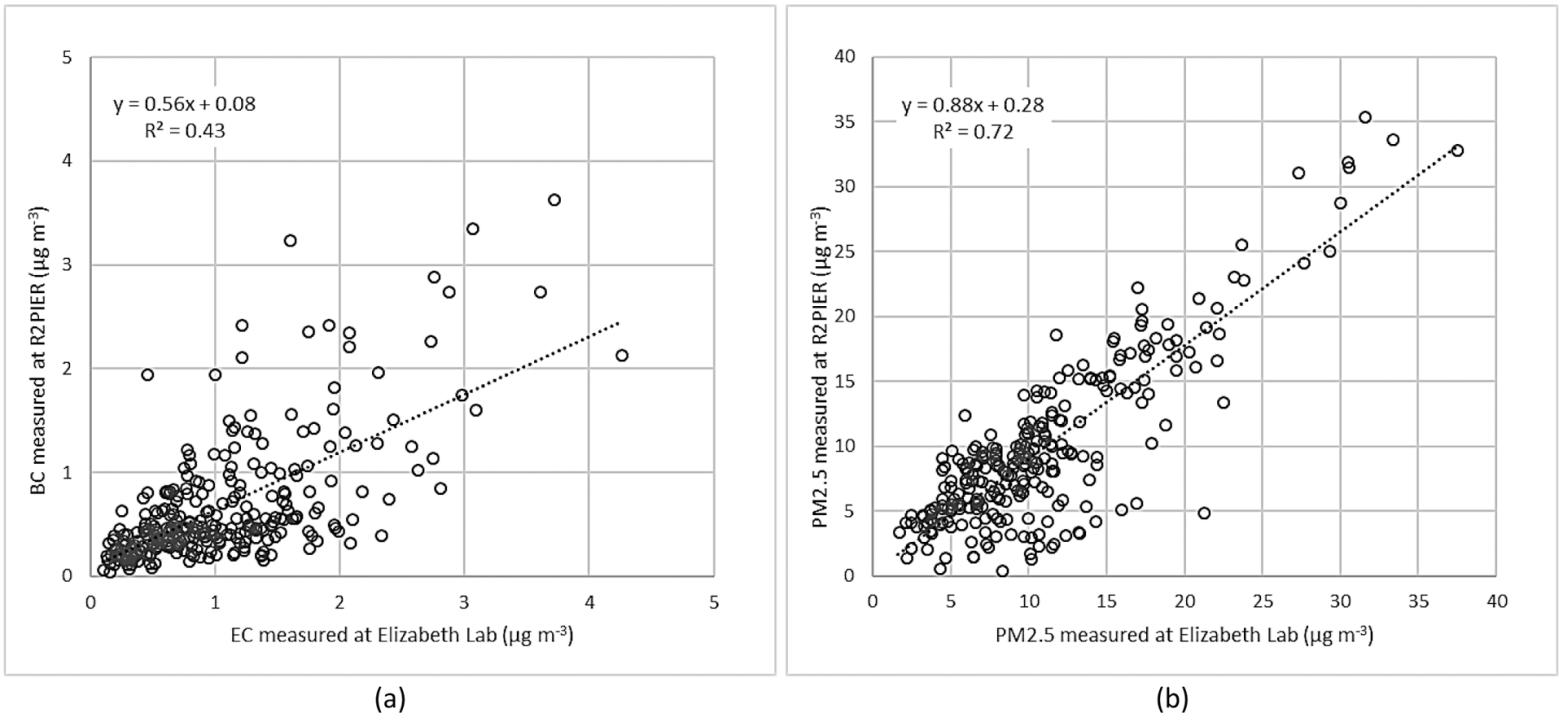
Comparison of 24-hour average (a) BC and (b) PM_2.5_ measurements at a nearby NJDEP site versus the R2PIER site.

The nearest regulatory monitoring station to the R2PIER site was New Jersey Department of Environmental Protection’s Elizabeth Lab Chemical Speciation Network (CSN) site, located approximately 2 km to the southwest (Latitude: 40.641440; Longitude: −74.208365). Whereas the R2PIER site measured PM_2.5_ continuously with the SHARP monitor that combined nephelometry and beta-attenuation, the Elizabeth Lab site measured PM_2.5_ using an integrated filter collection approach with subsequent laboratory analysis. The integrated PM_2.5_ filters were collected on a 1-in-3 day sampling schedule. Comparison of the particulate matter measurements were made between the two sites, with the R2PIER data reduced and averaged to match the Elizabeth Lab CSN site sampling schedule (Fig. 3). Despite the differences in the detection methods and distance, the two PM_2.5_ daily average values had moderate correlation (R^2^ = 0.72). Although the R2PIER daily PM_2.5_ concentrations were within 6% of that measured at the Elizabeth Lab site, the difference in the daily concentration between the two sites ranged fairly widely (relative standard deviation of 37%). The R2PIER BC concentration was compared against elemental carbon (EC) measured at Elizabeth Lab on the 1-in-3 day schedule. Both BC and EC are operationally defined methods, with BC measured optically and converted to a mass concentration, while EC is measured through a thermal-optical method that separates organic and elemental carbonaceous mass fractions. Both methods are commonly used and a past study intercomparing the two methods in the New York City area observed moderate correlation but with slopes ranging from 0.86 to 1.23 (Venkatachari *et al.*, 2006). When comparing BC at R2PIER and EC at Elizabeth Lab sites, the two methods had moderate correlation (R^2^ = 0.43), with BC concentrations lower than EC.

### 3.2 Temporal Analysis

In order to visualize the coupled effects of wind direction, day of week and time of day, two pollutants (BC and SO_2_) affected primarily by direct emissions were visualized in a polar annulus graphs oriented by wind direction and time (Fig. S3). For SO_2_, maximum concentrations occur during winds from the NE and SW, with highest values during morning periods. Lowest concentrations appear to occur on Sundays, during afternoon periods and with wind from the NW. BC has similar temporal trends, however appears to have higher concentrations associated with winds from the N and some periods of higher concentration under winds from the E/SE compared to SO_2_. Shipping activity data indicate a repeatable diurnal pattern with higher number of pings in the evening hours and lowest mid-day, and with weekends having lower activity than weekdays (Fig. S4). The general range of variation, however, is modest with less than 10% variation above/below the average.

**Fig. 4.**
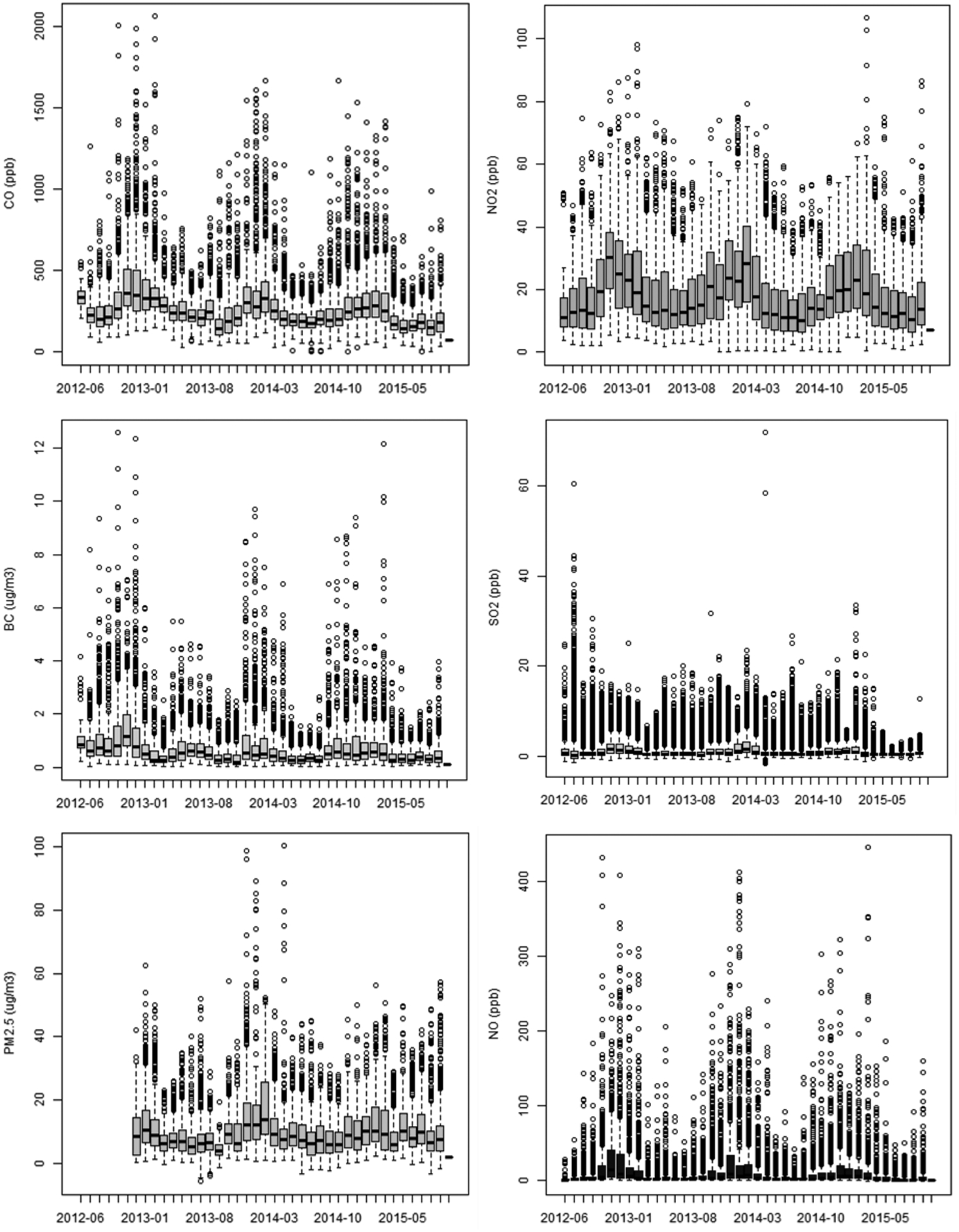
Box and whisker plots of hourly average concentrations by month during the sampling study.

**Fig. 5.**
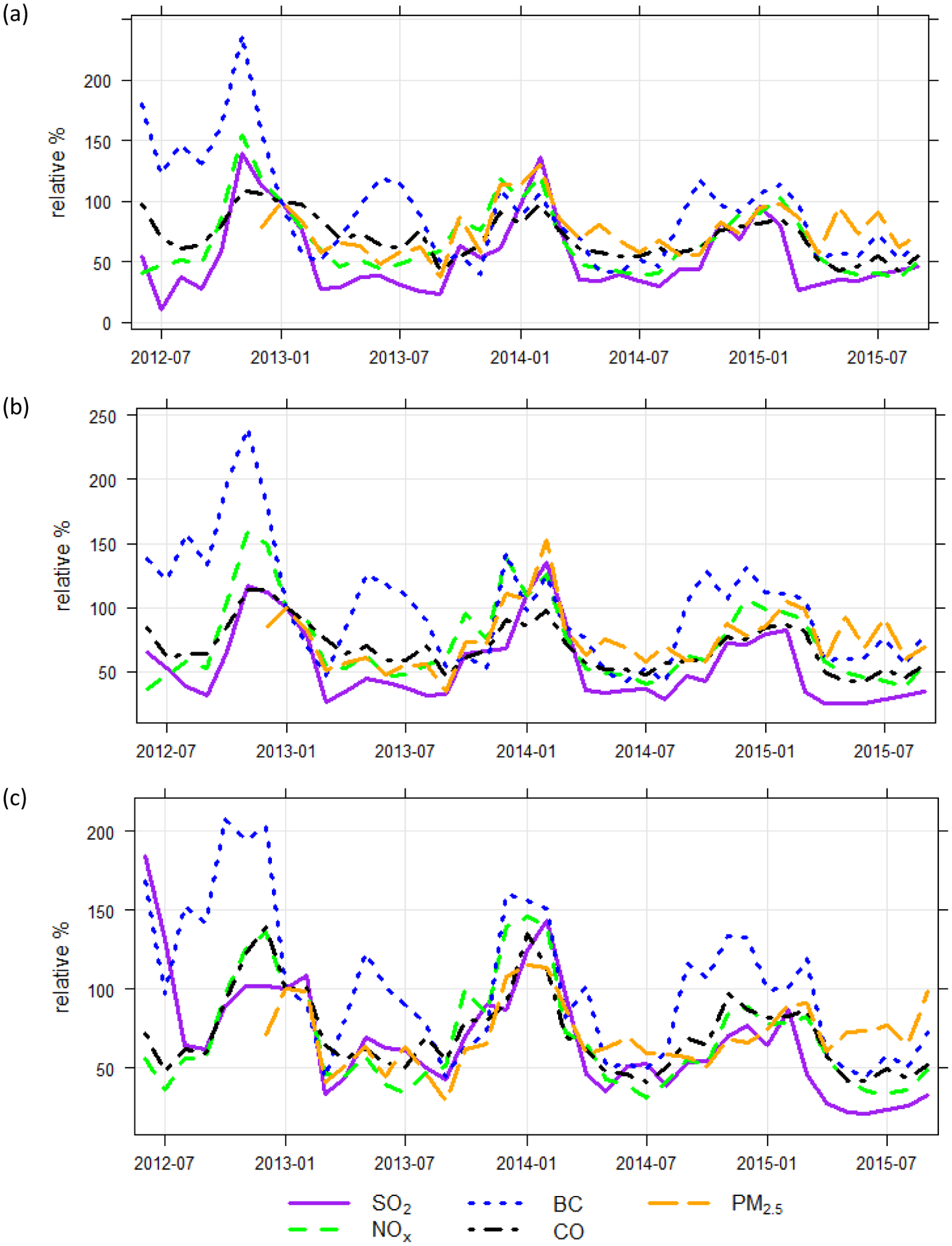
Normalized pollutant trends over 12/1/2012–9/30/2015 calculated as the monthly (a) median, (b) 75^th^ percentile, and (c) 95^th^ percentile. Each parameter was normalized to its respective 2013–01 concentration for ease of visualization.

Throughout the study period, the monitoring station exhibited variation in hourly concentrations (Fig. 4). The coefficient of variation (CV) was calculated by computing the standard deviation of the hourly averages divided by the mean of the averages. The CV was highest for NO (2.65), followed by SO_2_ (1.45), BC (1.21), PM_2.5_ (0.79), NO_2_ (0.65), and CO (0.60). In a near-source environment, the high concentration excursions of directly emitted pollutants would likely be related to proximate emissions. To explore how the overall concentrations varied over time, as well as the occurrence of high concentration episodes, monthly percentiles (50^th^, 75^th^, and 95^th^) were compared (Fig. 5), normalized to their respective January-2013 concentration for ease of visualization. BC stands out in having a slightly different seasonal signal in 2013, with higher concentrations in summer 2013 while other parameters had a local minima. For other periods, the general seasonal trends are similar across the pollutant types. SO_2_ stands out in both its decreased frequency of outliers (Fig. 4) and an associated decrease in the 95^th^ percentile, by approximately half, in spring/summer 2015 compared to the same time periods in 2013 and 2014. This shift is likely related to the introduction of lower sulfur fuel requirements which were enforceable in January 2015.

**Fig. 6.**
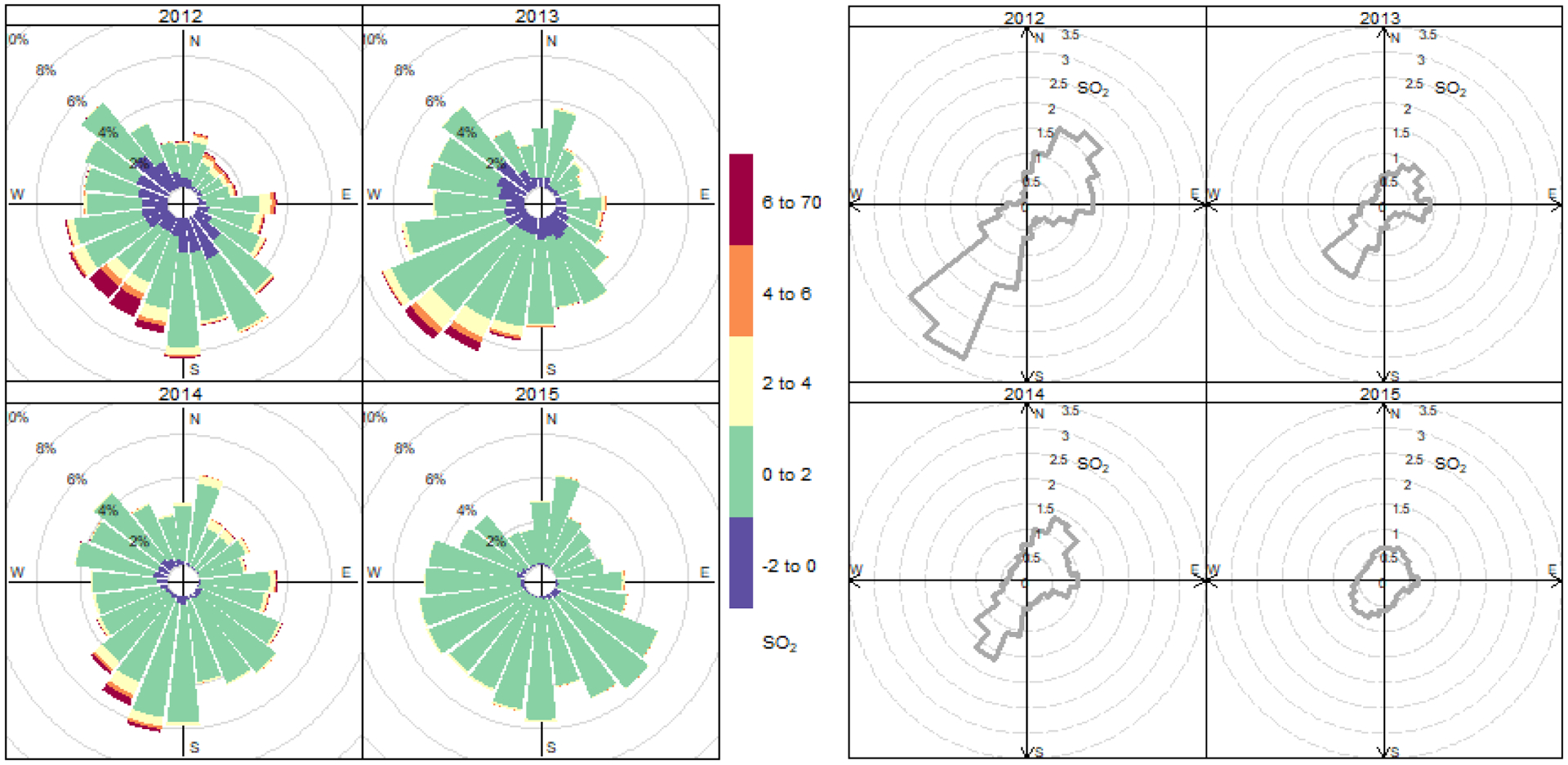
Pollution rose of SO_2_ (left) and calculated mean (right) in 15 degree wind direction bins covering the months of July, August and September measured between 2012–2015. Slight negative values are included as they represent near-zero observations; calm periods indicated by wind speeds below 0.5 m s^−1^ are excluded.

Pollution wind roses and wind-directional averages of SO_2_ (Fig. 6) for successive summer quarters (July through September) of the four monitored years reveal higher concentrations associated with winds from the southwest and northeast. Compared to earlier summers, the mean decreases from these two wind sectors in summer 2015 and shows little wind directionality. Comparing 2014 and 2015 and selecting for winds between 15 and 75 degrees (winds from the NE), mean concentrations from that sector were significantly lower by a factor of 0.55 with a Kruskal-Wallis test p-value < 0.01. For the SW wind sector, winds between 195–255 degrees, there was a significant decrease (p-value < 0.01) in mean SO_2_ by a factor of 0.60.

### 3.3 Nonparametric Trajectory Analysis

**Fig. 7.**
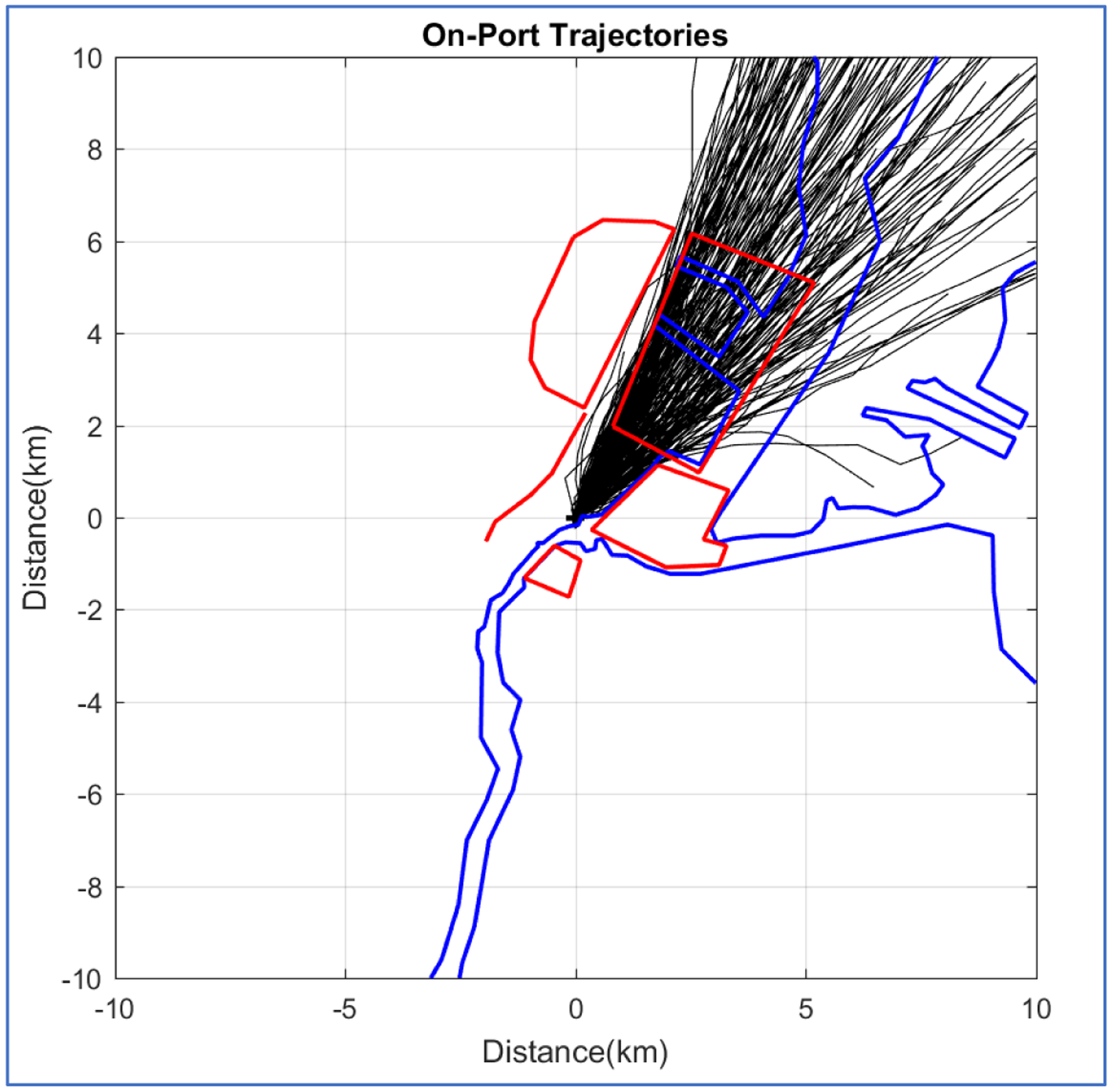
One-hour back-trajectories that pass directly over the Port to the monitoring site situated in the center of the map; every 500^th^ trajectory is shown. These trajectories include only those that pass over the Port and none of the other source areas outlined in red: the airport north of the site, nearby portion of the turnpike running north and west of the site, port area to the northeast of the site, waterway east of the site with ship traffic, and container terminal south of the site.

A major problem in assessing the impact of emissions changes in the Port is the presence of large nearby sources. Nonparametric Trajectory Analysis (NTA) was used to separate out the source areas for the purpose of sharpening the trends in the air quality data affected by mandated control strategies. The study period had a total of 1,623,765 one-hour back-trajectories computed for each minute and ending at the R2PIER site. Of these, 100,521 trajectories passed directly over the Port to the NE of the site (Fig. 7) while passing over no other known nearby source areas. Periods low wind speeds (calms) posed little difficulty for the trajectory calculations. Wind speeds at Newark airport (KEWR) are reported in whole knots and only 0.52% of the observations were less than 1 knot (0.514 m s^−1^). Wind speeds at the R2PIER site were reported to an accuracy of 0.1 m s^−1^ and only 0.16% of the observations were less than this.

**Fig. 8.**
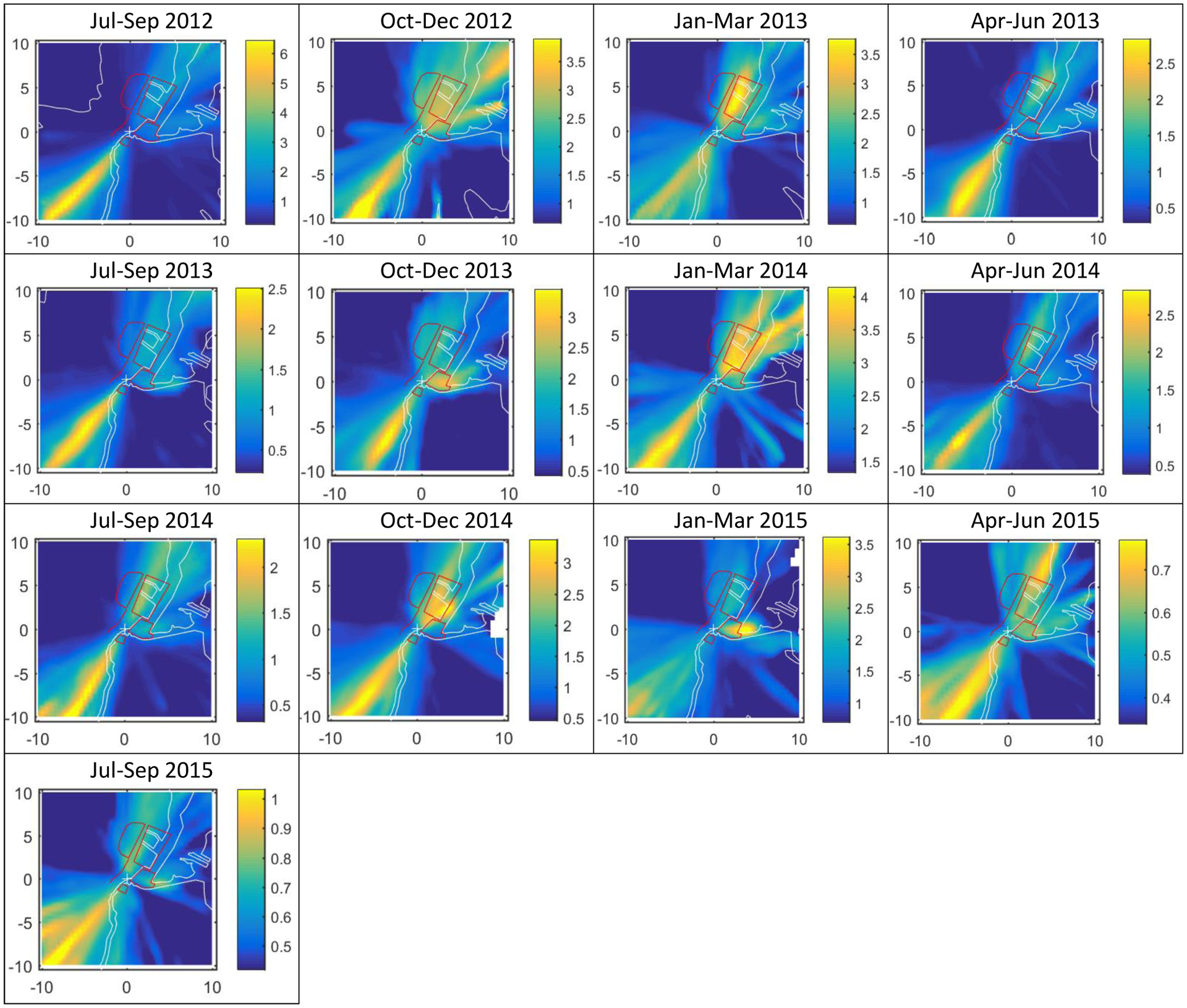
Nonparametric trajectory analysis results for sulfur dioxide, where the monitoring site is situated in the center of each map and the surrounding ±10 km area is shown. The colorbar represents expected concentrations at the monitoring site (ppb) when the air mass passed in a trajectory over the associated geographic area. Red outlines indicate nearby potential source areas, including the airport north of the site, nearby portion of the turnpike running north and west of the site, port area to the northeast of the site, waterway east of the site with ship traffic, and container terminal south of the site. The trajectories were placed on a 20 × 20 km grid with 500 m grid spacing to produce the NTA plots.

This technique allowed the impact of the Port and other source areas to be isolated for trend analysis. This high volume of trajectories revealed expected SO_2_ concentrations at the monitoring site on a quarterly basis as air masses passed over surrounding geographic areas (Fig. 8). The map visualizations show higher concentrations associated with areas to the southwest and northeast of the site, with several quarters showing higher source area contributions from the bay area due east of the monitoring site. BC and NO_x_ results (Figs. S5–S6) also indicate higher concentrations associated with areas to the northeast of the site, but relatively less from the southwest compared to the SO_2_ results. Specifically, the NTA maps show that the highest average concentrations at the R2PIER site were associated primarily with transport from the Port to the northeast and the container terminal to the southwest, and occasionally with transport from Newark Bay. The third quarter (July through September) maps show little year-to-year variation in geographic pattern, but a great reduction in maximum mixing ratio from 6 to 1 ppb.

Focusing on the subset of trajectories that pass solely over nearby geographic areas associated with ship traffic and cargo handling, Table 3 provides the change in concentration at the monitoring site for on-source trajectories comparing the last 5 quarters of the study (July 1, 2014–October 1, 2015) to the first 5 quarters of the study (July 1, 2012–October 1, 2013). These longer time spans were selected to provide a significant volume of trajectories to assess temporal shifts in source emission areas over the study period and use identical spans of months to minimize bias due to seasonality. This analysis indicates a decrease of 34–41%, 11–17%, and 28–41% for SO_2_, NO_x_, and BC, respectively, for air masses passing over three specific nearby source regions. An important caveat to these findings is that the concentrations input to the NTA analysis were absolute values and not background-subtracted. While the influence of background seasonality should be lessened by using the same 15 months for the “before” and “after” time windows and focusing on pollutants expected to be highly influenced by nearby emissions, additional research to separate the background component from the time series would further refine these estimates.

## 4 CONCLUSIONS

This study utilized research-grade and trace-level instrumentation to maximize the potential air quality insights from the data through careful siting and sustained collection of high time-resolution data. Comparison of the R2PIER site against a nearby regulatory monitoring site revealed correlation in measured PM_2.5_ and BC. Although TEUs increased and ship activity (vessel pings) did not markedly change, NTA analysis indicated decreases of SO_2_, NO_x_, and BC associated with port-related geographic areas when comparing the last 5 quarters measured to the first 5 quarters. The trends indicated by NTA analysis were directionally consistent with “port-wide” emission inventory changes over 2012–2015 for SO_2_ (PANYNJ, 2019b) and both analyses found relatively less change in NO_x_ although in differing directions. This comparison is limited, as this study’s NTA analysis emphasizes geographic areas surrounding the monitoring site position and the in situ monitoring data may be affected by sources outside the scope of the Port Authority’s inventory (e.g., emissions related to activities from privately-owned terminals).

As air monitoring rates increase with the evolution of air monitoring technology (e.g., Snyder *et al.*, 2013), retention and analysis of high time resolution air monitoring data may enable the isolation of local emission signals. Coupled measurements of directly emitted pollutants and wind, along with monitor siting to minimize collinearity in sources, increases the likelihood of resolving geographic source emission areas influencing the measurements and gaining insight into the impacts of emission reduction efforts.

## Supplementary Material

SI

## Figures and Tables

**Table 1.** Monitoring site instrumentation.

Measurement	Instrument	Time resolution
Carbon monoxide (CO)	Ecotech, Model EC9830T	1 min
Sulfur dioxide (SO_2_)	Ecotech, Model EC9850T	1 min
Oxides of nitrogen (NO, NO_2_, NO_x_)	Ecotech, Model EC9841T	1 min
Black carbon	Dual-channel Aethalometer, Model AE22, Magee Scientific	1 min
Fine particulate matter (PM_2.5_)	PM SHARP, Model 5030i, Thermo Scientific	1 min
Wind speed and direction	Ultrasonic anemometer, RM Young	1 min
Temp/RH	Vaisala, HMP45A	1 min

**Table 2.** Study wide averages and seasonal concentrations.

Year/Months	NO (ppb)		N0_2_ (ppb)		NO_x_ (ppb)		CO (ppb)		SO_2_ (ppb)		BC (μg m^−3^)		PM_2.5_ (μg m^−3^)		WS (m s^−1^)		T(°C)		RH (%)	
	Avg	Comp.^a^	Avg	Comp.	Avg	Comp.	Avg	Comp.	Avg	Comp.	Avg	Comp.	Avg	Comp.	Avg	Comp.	Avg	Comp.	Avg	Comp.
2012/JAS^b^	4.9	100%	15.9	100%	20.8	100%	231	100%	0.9	100%	0.90	100%	n/a	0%	2.9	100%	23.5	100%	65.2	100%
2012/OND	26.1	91%	25.7	91%	51.8	91%	386	91%	1.7	91%	1.33	88%	n/a	23%	2.9	91%	8.6	92%	68.6	92%
2013/JFM	13.0	100%	22.2	100%	35.2	100%	249	100%	1.2	100%	0.54	86%	10.7	94%	3.6	100%	2.3	100%	60.9	100%
2013/AMJ	4.4	100%	16.8	100%	21.2	100%	246	100%	0.8	100%	0.68	100%	7.5	100%	3.5	100%	16.4	100%	64.2	100%
2013/JAS	4.8	100%	16.5	100%	21.2	100%	222	100%	0.7	100%	0.55	100%	6.6	93%	2.9	100%	22.6	100%	63.9	100%
2013/OND	19.4	100%	23.1	100%	42.5	100%	269	73%	1.2	96%	0.62	95%	12.2	87%	3.2	100%	7.9	100%	68.7	100%
2014/JFM	20.0	100%	25.1	100%	45.1	100%	335	100%	1.9	100%	0.76	100%	14.4	90%	3.4	100%	−0.5	100%	58.3	100%
2014/AMJ	5.3	100%	15.5	100%	20.7	100%	208	100%	0.7	100%	0.41	100%	9.3	98%	3.3	100%	16.3	100%	62.2	100%
2014/JAS	5.2	100%	14.0	100%	19.1	100%	212	100%	0.8	100%	0.46	100%	7.7	100%	2.8	100%	22.2	100%	64.6	100%
2014/OND	14.7	100%	18.9	100%	33.6	100%	282	100%	1.1	100%	0.82	89%	8.9	100%	3.3	100%	6.9	100%	63.0	100%
2015/JFM	13.7	100%	23.6	100%	37.4	100%	306	94%	1.1	100%	0.78	90%	11.9	100%	3.4	100%	−2.3	100%	56.1	100%
2015/AMJ	3.9	100%	16.2	100%	20.0	100%	175	100%	0.5	100%	0.38	100%	9.8	100%	3.4	100%	15.5	100%	60.1	100%
2015/JAS	2.8	100%	15.0	100%	17.8	100%	181	100%	0.6	100%	0.44	97%	9.8	100%	2.9	100%	22.5	100%	60.7	100%
N total (days)	1179		1179		1179		1149		1175		1136		990		1179		1180		1180	
Study wide average	10.6		19.1		29.7		261.7		1.0		0.7		9.9		3.2		12.5		62.8	
Seasonal std. dev.	7.7		4.2		11.7		66.5		0.4		0.3		2.3		0.3		9.2		3.7	

a Completeness calculated on a seasonal basis.

b Months abbreviated into quarters - JFM = January, February, March; AMJ = April, May, June, JAS = July, August, September; OND = October, November, December.

**Table 3.** Difference in mean concentrations associated with trajectories passing directly over source geographic areas and arriving at the monitoring site, comparing the last 5 quarters to the first 5 quarters.

	ΔSO_2_ (%)	ΔNO_x_ (%)	ΔBC (%)
Port (NE of site)	−41.05	−11.46	−27.82
Bay (E of site)	−34.34	−13.54	−41.28
Terminal (S of site)	−37.32	−16.79	−37.54
