# Supplementary material for: Three Years of High Time-resolution Air Pollution Monitoring in the Complex Multi-source Harbor of New York and New Jersey
Source: Aerosol Air Qual Res. Author manuscript; Available in PMC 2021 Feb 24. (PMC7903559; doi:10.4209/aaqr.2020.02.0069)
Supplement: SI [file NIHMS1667473-supplement-SI.pdf]

Supplemental Information:

Table S1. Metals data measured at R2PIER site (in ng m^{-3})

	S	K	Ca	Ti	V	Mn	Fe	Ni	Cu	Zn	As	Br	Cd	Ba	Hg	Rb	Sr
Mean	547	108	17.9	2.81	1.32	1.41	71.3	1.38	9.85	13.8	0.15	0.22	2.68	4.66	2.00	2.71	0.24
Standard deviation	376	40	23	3.42	3.2	2.4	100	3.2	20	58	0.08	0.35	1.89	2.38	3.40	3.01	0.14
CV	0.69	0.37	1.28	1.22	2.44	1.73	1.40	2.34	1.99	4.21	0.49	1.58	0.71	0.51	1.70	1.11	0.59

Table S2. Metals data comparison between R2PIER and Elizabeth Lab sites

	V (ng m^{-3})		Ni (ng m^{-3})	
	R2PIER	Elizabeth Lab	R2PIER	Elizabeth Lab
Average	1.25	1.19	1.45	1.82
Standard deviation	1.74	1.96	2.02	2.68
Max	8.35	8.6	10.5	11

[a]Daily values for 28 matching days over 6/2014 - 3/2015

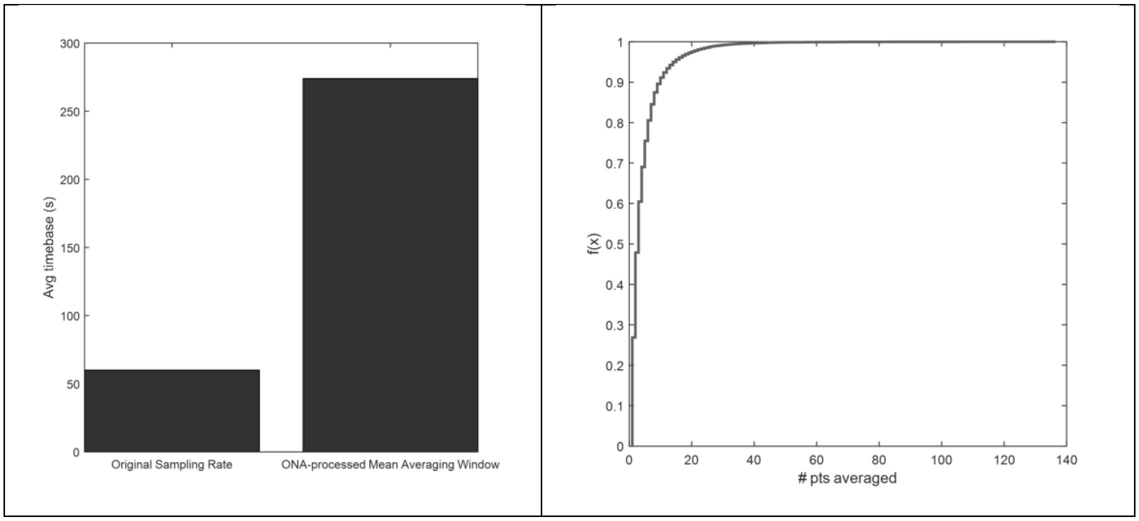

Fig. S1. Post-processing of BC data with the ONA algorithm, with the average time base shown on the left and empirical cumulative density function (ECDF) shown on the right.

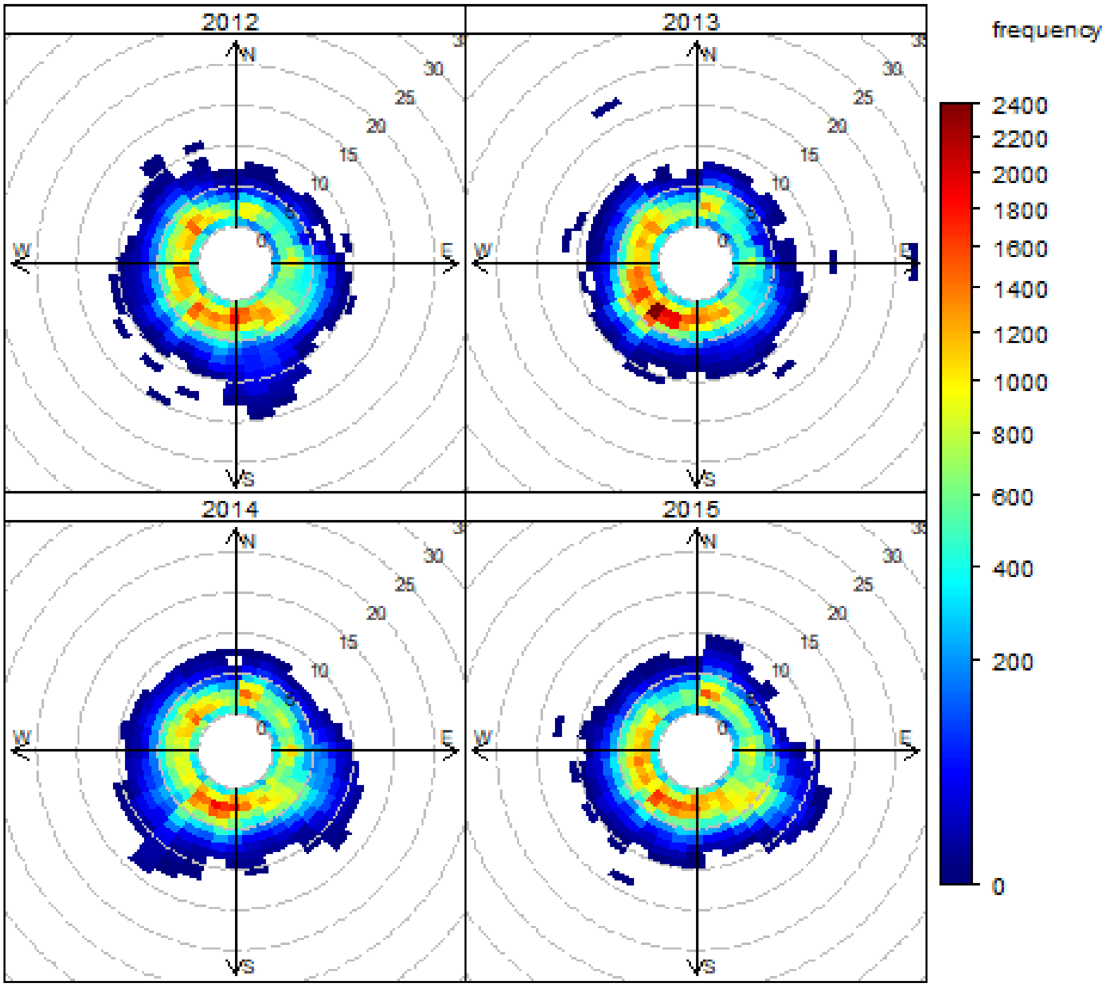

Fig. S2 Frequency of observations for a three-month window (July through September) by wind speed, wind direction, and year of sampling.

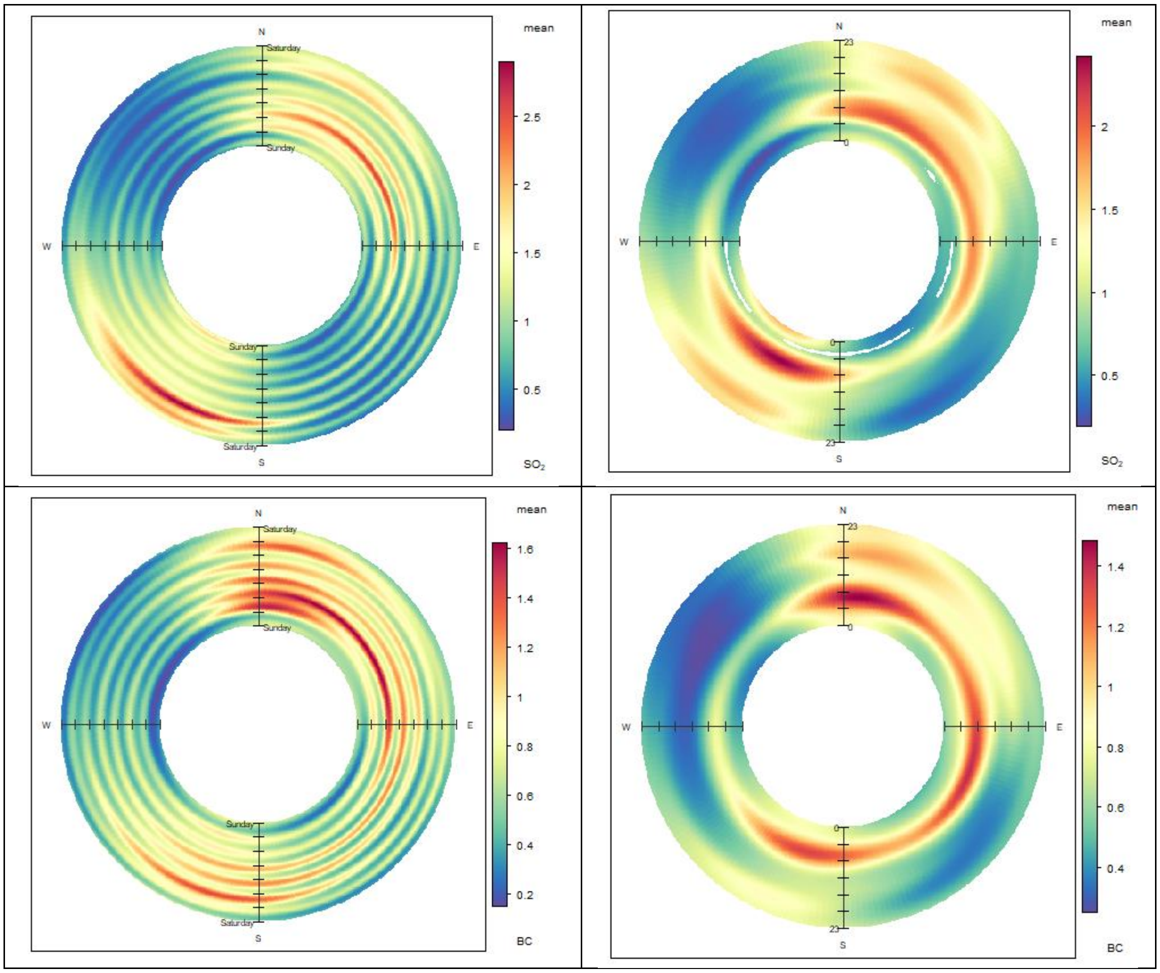

Fig. S3. Polar trends showing weekday (left) and hour of day (right) variation in mean concentration levels of SO_2 (top) and BC (bottom), oriented by wind direction. For the weekday plots, time proceeds from hours 0-23 for each day from the inner to outer circle (i.e., innermost point is hour 0 on Sunday, outermost point is hour 23 on Saturday).

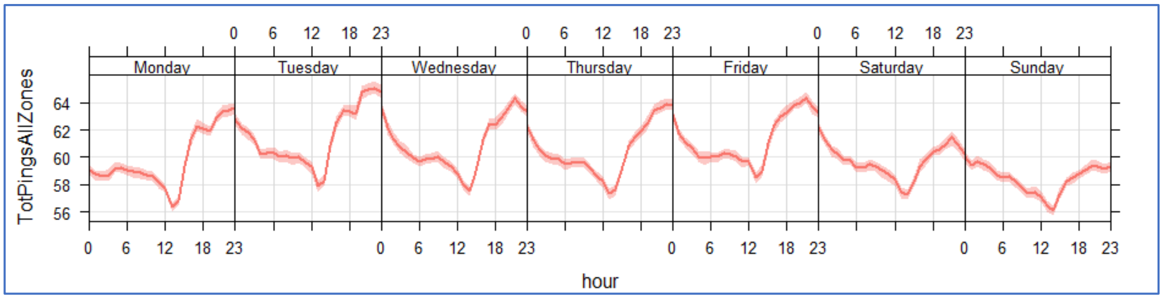

Fig. S4. Average number of pings from nearby ships during the study period, by hour of day and day of week

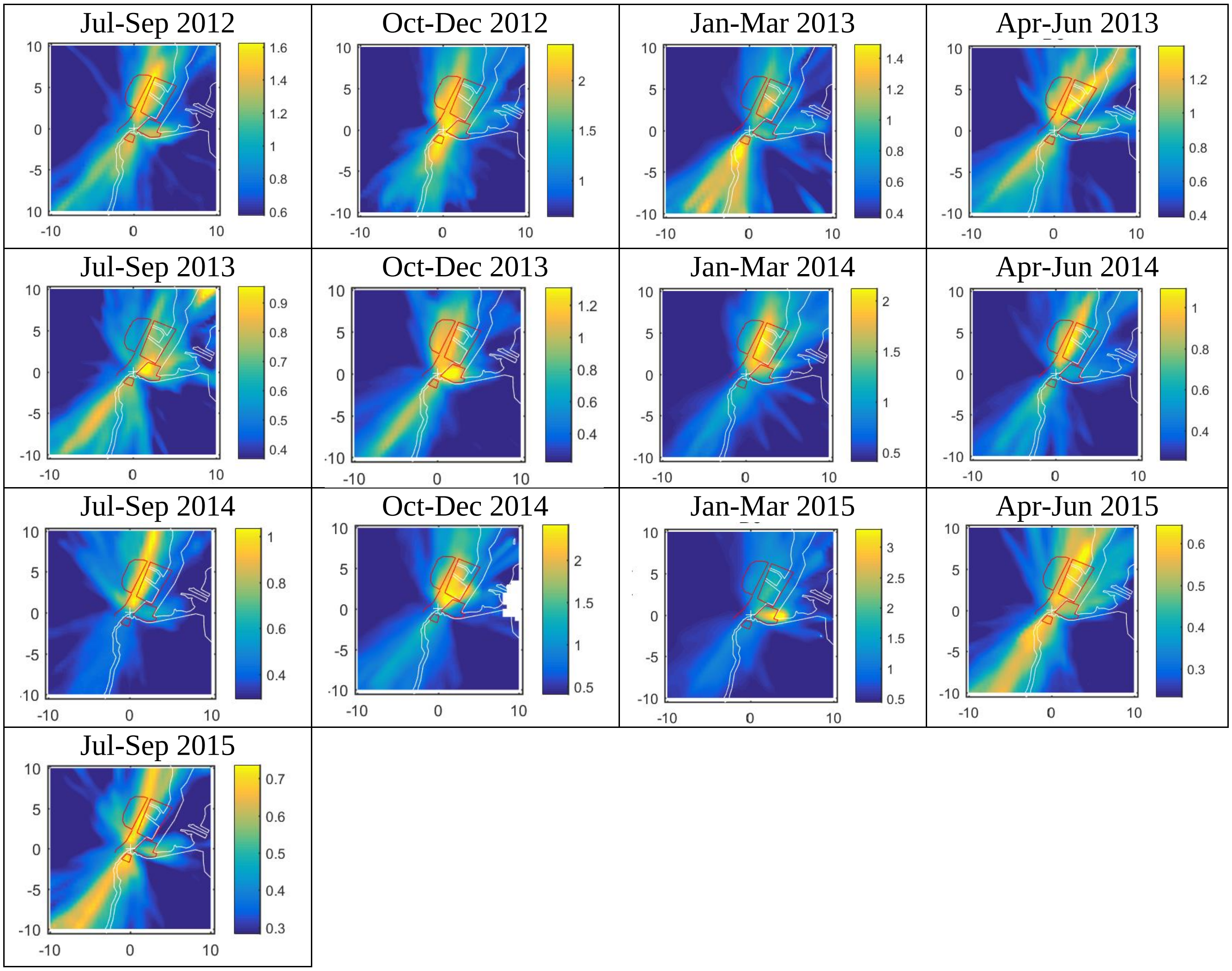

Fig. S5. Nonparametric trajectory analysis results for black carbon, where the monitoring site is situated in the center of each map and the surrounding +/- 10 km area is shown. The colorbar represents expected concentrations at the monitoring site (µg m^{-3}) when the air mass passed in a trajectory over the associated geographic area. Red outlines indicate nearby potential source areas, including the airport north of the site, nearby portion of the turnpike running north and west of the site, port area to the northeast of the site, waterway east of the site with ship traffic, and container terminal south of the site.

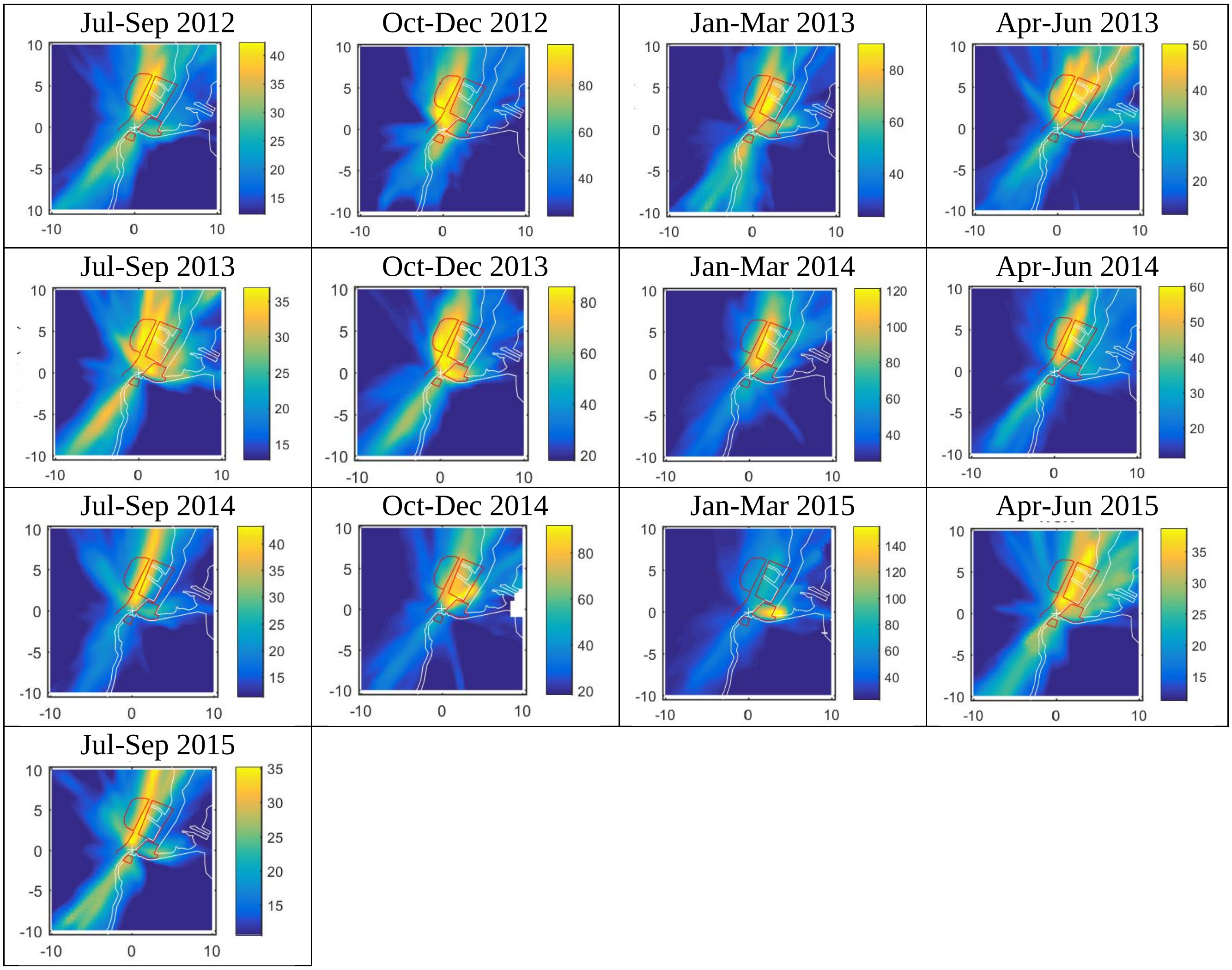

Fig. S6. Nonparametric trajectory analysis results for NOx, where the monitoring site is situated in the center of each map and the surrounding +/- 10 km area is shown. The colorbar represents expected concentrations at the monitoring site (ppb) when the air mass passed in a trajectory over the associated geographic area. Red outlines indicate nearby potential source areas, including the airport north of the site, nearby portion of the turnpike running north and west of the site, port area to the northeast of the site, waterway east of the site with ship traffic, and container terminal south of the site.
